# The Role of Micronutrients in Human Papillomavirus Infection, Cervical Dysplasia, and Neoplasm

**DOI:** 10.3390/healthcare11111652

**Published:** 2023-06-05

**Authors:** Filippo Alberto Ferrari, Francesca Magni, Mariachiara Bosco, Giulia Biancotto, Pier Carlo Zorzato, Antonio Simone Laganà, Vito Chiantera, Ricciarda Raffaelli, Massimo Franchi, Stefano Uccella, Simone Garzon

**Affiliations:** 1 Department of Obstetrics and Gynecology, AOUI Verona, University of Verona, 37126 Verona, Italy; 2 Unit of Gynecologic Oncology, ARNAS “Civico-Di Cristina-Benfratelli”, Department of Health Promotion, Mother and Child Care, Internal Medicine and Medical Specialties (PROMISE), University of Palermo, 90127 Palermo, Italy

**Keywords:** human papillomavirus, cervical dysplasia, cervical cancer, micronutrients, cancer prevention

## Abstract

There is evidence that diet and nutrition are modifiable risk factors for several cancers. In recent years, attention paid to micronutrients in gynecology has increased, especially regarding Human papillomavirus (HPV) infection. We performed a review of the literature up until December 2022, aiming to clarify the effects of micronutrients, minerals, and vitamins on the history of HPV infection and the development of cervical cancer. We included studies having as their primary objective the evaluation of dietary supplements, in particular calcium; zinc; iron; selenium; carotenoids; and vitamins A, B12, C, D, E, and K. Different oligo-elements and micronutrients demonstrated a potential protective role against cervical cancer by intervening in different stages of the natural history of HPV infection, development of cervical dysplasia, and invasive disease. Healthcare providers should be aware of and incorporate the literature evidence in counseling, although the low quality of evidence provided by available studies recommends further well-designed investigations to give clear indications for clinical practice.

## 1. Introduction

Papillomaviruses are double-stranded DNA viruses with a particular tropism for squamous epithelium. More than 200 human papillomaviruses (HPVs) have been identified and classified in “high-risk HPV”(HR-HPV) and “low-risk HPV” types according to their cancerogenic potential [1]. HPV infections are associated with different types of lesions, such as benign proliferative lesions (warts), as well as precursor lesions with the ability to progress to high-grade neoplasia and invasive cancer, including cervical, anal, penile, vulvar, vaginal, and oropharyngeal tumors [1,2].

Cervical cancer (CC) is a significant public health issue affecting women worldwide, with approximately 565,000 new cases and 280,000 deaths annually [3]. A consistent association between HR-HPVs and CC has been established, and persistent infection is the main etiological factor of CC [4]. The International Agency for Research on Cancer (IARC) classified HPV 16, 18, 31, 33, 35,45, 51, 52, 56, 58, 59, 66, and 68 as HR-HPVs, being found in 99.7% of CCs [5]. In the cervical tissue, HPV infection can induce various degrees of premalignant changes called cervical intraepithelial neoplasia (CIN). CIN lesions include low-grade cervical dysplasia (LSIL/CIN1), moderate dysplasia (CIN2), and severe dysplasia/carcinoma in situ (CIN3/CIS) [6]. However, only a small percentage of infected women progress to invasive disease, and more than 90% of HPV infections are transient and regress within 6–12 months after their onset [7]. Only 5–10% of women develop non-invasive squamous intraepithelial lesions, and less than 1% of infected women will develop invasive CC, indicating that, beyond HR-HPVs, other biological factors play a pivotal role [6]. While CC screening has helped to reduce the incidence and mortality of CC in conjunction with the HPV vaccine, which also demonstrated a therapeutic role, there is growing interest in the role of diet and nutrition in cancer prevention [8,9].

Micronutrients (essential trace elements and vitamins) demonstrated a possible role in optimizing health and preventing or treating diseases [10,11]. Recently, the knowledge and understanding of the biochemical functions of these nutrients and their pivotal role in the maintenance of tissue function increased [12,13]. Different in vitro studies demonstrated the essential effect on all aspects of the immune system, suggesting that impaired immune function and increased risk of infection may be influenced by subclinical deficiencies of trace elements and vitamins [12]. Most micronutrients provide an antioxidant activity that scavenges free radicals and prevents DNA damage. Others additionally are genomic regulators, influencing methylation reactions, reducing immunosuppression, and stabilizing the p53 protein. In vitro, antioxidants show potential capacity for the modulation of carcinogenic processes [14]. In recent decades, different studies have focused on the relationship between dietary intake, serum element levels, and the development of tumors, including CC [14,15,16].

Based on the growing interest in the role of diet and nutrition in cancer prevention and the burden of HPV infection and CC, this review aimed to summarize all available scientific evidence about the role of micronutrients, vitamins, and minerals on HPV infection, its progression to cervical intraepithelial (CIN) lesions, and invasive CC development to clarify what is known and what needs further investigation.

## 2. Materials and Methods

### 2.1. Study Design and Search Strategy

We systematically reviewed the literature by performing a literature search in the electronic databases Scopus, PubMed/MEDLINE, and ScienceDirect, from inception of the databases to 31 December 2022. A combination of the following keywords was used: “antioxidants”, “vitamins”, “micronutrients”, “minerals”, ”vitamin A”, “vitamin B”, “vitamin C”, “vitamin D”, “vitamin E”, “vitamin K”, “calcium”, “folate”, ”retinol”, “carotenoids”, ”dietary intake”, “micronutrients supplementation”, “cervical intraepithelial neoplasia”, “uterine cervical dysplasia”, “cervical cancer”, and “human papillomavirus infection”.

### 2.2. Inclusion Criteria

We included only clinical studies investigating the association between HPV infection, persistent HPV infection, CIN presence, development or progression, or CC and any micronutrient, vitamins, and minerals of interest (calcium; zinc; iron; selenium; carotenoids; vitamins A, B12, C, D, E, and K). Case-control or cohort studies and clinical trials were all eligible. Case reports, case series with less than 5 patients, reviews, and in vitro or animal studies were excluded.

### 2.3. Study Selection

Two authors (FFA, FM) independently screened titles and abstracts following inclusion and exclusion criteria. Two other authors double-checked selected studies and provided clinical and scientifical experience (SG, MCB). Any disagreement over the eligibility of the studies was resolved through discussion with a fifth author (SU).

### 2.4. Data Extraction and Data Synthesis

A standardized form was used to extract data from the included studies. The data extracted were evaluated by analyzing the following features: authors; country; year of publication; studied micronutrient; study design; serum, tissue levels or daily dietary intake of micronutrient; number of women enrolled in the study; and data about HPV infection, CIN, and CC in included patients.

## 3. Results

**Figure 1:**
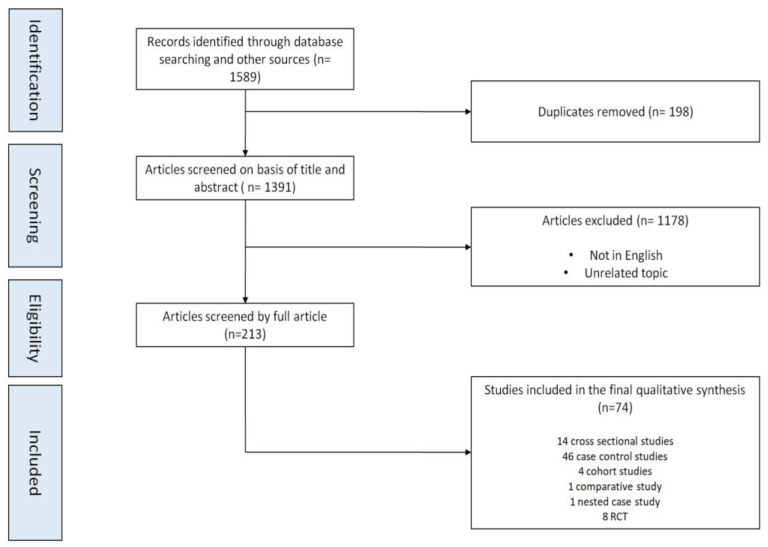
Flow diagram of the selection of the studies.

We identified 1589 manuscripts with the initial systematic literature search. After duplicate removal and title and abstract screening, we revised the full text of 213 manuscripts. Finally, we included in the final synthesis 74 papers (Figure 1). Due to significant heterogeneity between studies, quantitative data synthesis was not possible; hence, we performed a qualitative synthesis, organized in thematic sections, to summarize the available evidence.

### 3.1. Calcium

Calcium (Ca) is a trace element essential in cell division, cell proliferative processes, neoplastic transformation, and immune response [17]. Previous studies demonstrated that dietary calcium could regulate oxidative stress by modulating calcium transport and signaling [18,19]. Moreover, animal studies suggested a correlation between dietary calcium restriction and oxidative stress, with a preventive effect of Ca intake on oxidative and inflammatory stress [20,21]. On the other hand, in vitro evidence showed an association between high extracellular Ca levels and the persistence or progression of HPV infection [22,23]. In agreement with this thesis, Sengupta et al. found significantly higher serum Ca levels in patients with CC than in controls [24].These results disagree with the findings of Hwanget et al., who reported that adequate dietary calcium (OR, 0.21; 95% CI, 0.08–0.50) was significantly associated with a lower risk of CIN grade 2–3 [25] (Table S1). Instead, Liu et al. found no association between Ca intake and cervical dysplasia, with a multivariate-adjusted OR of 0.9 (95% CI 0.4–2.1) for the highest versus the lowest level of intake [26]. A recent cross-sectional study using data from the National Health and Nutrition Examination Survey (NHANES) investigated the correlation between dietary Ca and HPV infection in American females. The authors demonstrated no linear correlation between dietary calcium intake and HPV infection. However, after converting the dietary calcium amounts using the log2 function, a non-linear correlation was detected, and a protective effect of increased Ca intake was reported in a specific range. They found a saturated effect of daily intake and HPV infection (Table S1) [27].

To summarize, evidence regarding the correlation between Ca and the risk of CC is controversial. In vitro, higher extracellular calcium levels have been associated with the persistence of HPV infection [22,23]. However, evidence from human studies demonstrated that adequate dietary Ca intake might have a protective role, while one of the included studies found no association between Ca intake and the risk of cervical dysplasia [25,27]. Therefore, higher-quality studies and more robust evidence are needed before conclusions regarding the association between dietary calcium and the risk of CC can be drawn.

### 3.2. Zinc

Zinc is an essential micronutrient traceable in different tissues. It regulates immune function, DNA synthesis, RNA transcription, reduction of oxidative stress, cell aging, and apoptosis [28]. It acts as a cofactor of many enzymes and is a cellular growth protector. Several studies demonstrated that zinc might modify viral functions and inhibit replication [29,30]. Moreover, it has been shown to induce apoptosis in malignant cells in prostate cancer [31], ovarian cancer [32], and colon cancer [33].

Relevant studies are summarized in Table S2. Grail et al. reported a decreased zinc serum level in CIN 1 and invasive CC [34], consistent with the results of an Indian study in which zinc was significantly lower in CC compared with healthy controls (*p* < 0.001) [35]. Furthermore, Cunzhi et al. reported decreased serum and lower zinc levels in CC tissue [36]. In contrast, findings from a case–control study of 99 cases of CC demonstrated a reduced plasma level only in stages III and IV and in recurrent disease, without any statistical association for pre-invasive lesions or early stages [37].

The effect of intravaginal zinc on HPV infection persistence was evaluated in a Korean comparative study [38]. The topical administration of zinc citrate solution twice a week for three months demonstrated a higher rate of HPV clearance (64.47% vs. 25.51%; *p* < 0.001), and a decreased risk of persistent HPV (OR 0.079, 95% CI 0.039–0.165; *p* < 0.001). Moreover, at the end of the treatment, a higher percentage of intraepithelial lesions regressed in the zinc arm, especially in the LSIL group [38]. The impact of daily dietary intake was investigated by Barchitta et al., with the study showing a lower OR for infection with HR-HPVs (OR = 0.46; 95% CI = 0.27–0.80; *p* = 0.006) [39]. Data from 4628 women were obtained from the National Health and Nutrition Examination Survey (NHANES) [40]. The highest quartile of zinc intake showed an adjusted odds ratio for HR-HPVs infection of 0.72 (95% CI, 0.54–0.98) compared to the lowest quartile [40]. Recently, Ayatollahi et al. performed a randomized controlled clinical trial among 40 patients with an HPV-positive test, ASCUS, or LSIL, to evaluate the effect of 220 mg of zinc every 12 h on the clearance of HPV infection. After three months, zinc supplementation was demonstrated to reduce the risk of persistence of infection (OR 0.130, CI 95% 0.04–0.381; *p* < 0.001) and progression (OR 0.301, 95% CI 0.777–0.116; *p* = 0.012) from the baseline cytology [41].

To summarize, the studies mentioned above demonstrate that reduced serum zinc levels can be found in patients with LSIL and CC [34,36], mostly at advanced stages [38]. Moreover, evidence shows that higher dietary and intravaginal zinc supplementation may be associated with a reduced risk of HR-HPV infections and persistence [39,40,42,43].

### 3.3. Iron

Iron is a trace element necessary for the normal functioning of proteins and enzymes. Iron-containing proteins are required for cell respiration, oxygen sensing, oxygen transport, oxygen metabolism, energy metabolism, DNA synthesis, and repair and signaling [42].

Ito et al. found that, among 98 patients with untreated CC, 50 (51%) had elevated levels of serum ferritin [43] (Table S3). Out of 36 patients who were then surgically treated, those who had above- average pre-operative ferritin levels were more likely to show parametrial invasion or lymph node metastasis compared to women with pre-operative ferritin levels within the normal range (12/16, 75% vs. 3/20, 15%) [43]. Moreover, the authors found that ferritin levels tended to decrease to the normal range four weeks after treatment in many patients. In contrast, the development of elevated levels was closely associated with a poor prognosis [43]. A more recent American observational study noted that women in the highest category of ferritin levels were less likely to show a regression of oncogenic HPV infections compared to women with lower ferritin levels (adjusted HR = 0.73; 95% CI 0.55–0.96) [44]. This association was stronger among women with enriched iron stores (≥120 μg/L). On the other hand, no significant association was found between ferritin at adequate or increased levels and the clearance of non-oncogenic HPV infections [44]. The authors of this study hypothesized that rising iron stores could increase the risk of persistent HPV infection by promoting viral replication and transcription and contributing to oxidative DNA damage [44]. Lastly, a Chinese case–control study investigated the relationship between trace elements and the incidence of CC. It concluded that iron concentration in CC tissue was significantly higher than in paired non-lesion tissue (*p* < 0.005). Moreover, the iron serum concentration was found to be significantly lower in patients with CC compared to healthy subjects (*p* < 0.001) [36].

To summarize, evidence regarding the correlation between iron levels and CC is inconclusive. Higher serum levels of ferritin have been found in patients with untreated CC, and higher ferritin levels have been associated with a poorer prognosis [43,45] and, in another study, with reduced regression in the case of HR-HPV infections [46]. On the other hand, results from different research cases show reduced iron serum levels in patients with CC compared to controls [37]. Therefore, no clinical conclusions can be drawn from the present literature, and more quality studies are needed, possibly investigating the possible effect of dietary calcium on the prevention or progression/regression of HPV infections.

### 3.4. Selenium

Selenium (Se) is an essential trace element that plays important functions in the human organism, an organism mediated by selenium-containing proteins (selenoproteins). Some selenium-mediated functions are related to the antioxidant system, thyroid hormone metabolism, and immune system [45]. Selenium exerts antioxidant activity in different ways, for example, by scavenging the reactive oxygen species (ROS) and improving the synthesis of enzymatic antioxidant glutathione peroxidase [46]. Moreover, there is evidence that Se carries antiviral activity [47].

Relevant studies are summarized in Table S4. Decades ago, Sundstrom et al. reported significantly lower Se serum levels in CC patients compared to controls (0.97 ± 0.06 μmol/L vs.1.26 ± 0.03 μmol/L, *p* < 0.001) [48]. Subsequently, Cunzhi et al. found a significantly lower concentration of Se in malignant tissues and serum of patients with CC compared to controls (*p* < 0.001) [36]. Subramanyam et al. confirmed the results reported from the studies mentioned above and found that chemotherapy treatment in patients with CC was associated with a significant increase in serum Se levels and, consequently, in antioxidant activity [49]. Instead, a recent case–control study found no significant difference in the mean serum selenium levels between cases and controls. However, a statistically significant difference in the mean value of serum selenium was observed between controls and histological subgroups within cases (CIN 1, CIN 2, and CIN 3) using the ANOVA test (*p* = 0.021) and between controls and CIN 3 patients (*p*-value = 0.016) [50]. On the contrary, data from the National Health and Nutrition Examination Survey (NHANES) found no significant association between dietary intake of Se and high-risk HPV infection [40]. To investigate the preventive activity of Se against the progression of cervical dysplasia, a recent randomized, double-blind, controlled trial compared the regression of the CIN 1 rate between 26 cases supplemented with 200 mg of Se and a control group treated with a placebo. Se supplementation for six months was associated with a significantly higher CIN 1 regression rate (88% vs. 56% in the placebo group, *p* = 0.01) [51].

To summarize the cited evidence, lower selenium serum levels have been found in cases with CC [50], and increased levels have been noticed after treatment [51]. Moreover, with the increasing severity of CIN, a significant reduction in selenium serum levels has been reported [52]. Interestingly, a RCT found that selenium supplementation is associated with a higher CIN1 regression rate [53]. However, data from the NHANES database found no association between dietary intake and HR-HPV infections [42].

### 3.5. Folate and Vitamin B12

Folate is a soluble vitamin and, together with vitamin B12, participates as a cofactor in many biological processes. Both are involved in homocysteine metabolism, are precursors of purine and pyrimidine basis in DNA synthesis, and are involved in DNA methylation [52]. The interaction with 5-methyltetrahydrofolate is crucial for methylation status, demonstrating a pivotal role in cancerogenic development [53]. Previous studies showed an inverse relationship between folate status and breast, colon, stomach, esophagus, and pancreas cancer risk. The study by Popescu et al. demonstrated that a possible site of HPV integration is a host chromosomal region sensitive to folate deficiency, suggesting a possible correlation between folate concentrations and the risk of cervical dysplasia and cancer [53]. Relevant studies are summarized in Table S5. Hernandez et al. published a case–control study on 431 women reporting an inverse association between total folate intake and premalignant cervical lesions, especially for HSILs [54]. Wang et al. evaluated the association also in the case of CC, reporting significantly lower dietary intake in cases than in controls. Still, the adjusted risk analysis showed no statistical significance [55]. However, they reported lower serum folate in cases compared to controls, with a significantly increased risk of CC with reducing plasma levels (*p* < 0.001) [56]. In a large Chinese cross-sectional study, Zhao et al. confirmed lower folate serum levels in HSILs compared to LSILSs and controls (*p* = 0.02) [57]. A study conducted in Turkey reported that in all cervical dysplasia groups, folate levels were lower in HPV-positive women than in negative ones [52]. Piyathilake et al. demonstrated that women with higher serum folate levels (>6 ng/mL) were at a lower risk of HR-HPV positivity compared to those with serum folate levels ≤ 6 ng/mL [58,59]. Subsequently, the same authors analyzed the influence of plasma concentrations of folate on the degree of HPV 16 methylation, showing that women with higher plasma levels and higher methylation were less likely to develop CIN2 or higher dysplastic lesions (*p* = 0.02) [60]. The same protective effect was also demonstrated for a high plasma concentration of vitamin B12, suggesting that improving vitamin B12 status and folate status may also be essential to maximize the protective effect [60]. Raghasuda et al. showed an increased risk of LSILs and CC in women with reduced serum levels of Vitamin B12 compared to controls (LSIL: OR 10.6; 95% CI = 4.11 to 27.6; *p* < 0.001; CC: OR 10.4; 95% CI = 3.88 to 27.9; *p* < 0.001) [61], consistent with the findings of Kwanbunjan et al. [62]. In contrast, the results of other authors did not find any significant association between vitamin B12 levels and HPV infection or related lesions [52,63]. The relationship between folate and the Fragile histidine triad (FHIT), a tumor suppressor gene frequently silenced in CC, was studied by Li et al. They found folate may sustain apoptosis and regulate FHIT gene methylation, possibly inhibiting cervical cell proliferation [64]. Consistently, Bai et al. confirmed the association of CC and CIN with folate deficiency (OR: 1.68 with serum level < 3.19 ng/mL), FHIT hypermethylation (OR: 11.47), and HPV HR infection (OR:4.63) with the progression of cervical lesions [65]. An Iran double-blinded randomized controlled trial investigated the role of a 5 mg/day supplementation for six months in a small cohort of women diagnosed with CIN 1. The results showed a greater percentage of lesion regression in the folate arm compared to the placebo group (83.3% vs. 52.0%, *p* = 0.019) [66]. Nonetheless, Sabihi et al. recently performed a randomized double-blinded controlled trial that reported an insignificant decrease in the recurrence of CIN 2–3 in patients supplemented with the same amount of daily folate for 12 weeks (3.3% vs. 16.7%, *p* = 0.08) [67].

To summarize, consistent evidence supports the association between low folate and vitamin B12 levels and an increased risk of premalignant cervical lesions and CC [58,59]. Similarly, high serum folate levels are associated with a reduced risk of HR-HPV infection positivity [62,63] and a reduced risk of developing CIN 2 or higher dyplasia [64,68]. However, other studies found no relationship between folate level and the risk of CC [54]. To conclude, two RCTs found that folate supplementation is associated with a significantly higher CIN 1 regression rate [69], but no significant decrease was found for the risk of recurrence of CIN 2/CIN 3 [70].

### 3.6. Carotenoids

Carotenoids are lipid-soluble micronutrients with high antioxidant activity and modulatory effects on immunity [71]. Moreover, they are involved in pathways involving cell growth and death, which is considered one of the major mechanisms through which they are implicated in cancerogenic processes [72]. More than five hundred carotenoids were identified, and some may be able to be converted into vitamin A. Lycopene, β-carotene, α-carotene, lutein, zeaxanthin, and β-cryptoxanthin are the most common carotenoids in human serum. Carotenoids can be schematically classified into oxygen-free carotenes (OFC; α-carotene, β-carotene, and lycopene) and oxygen-containing xanthophylls (OCX; zeaxanthin/lutein, neoxanthin, and fucoxanthin). Zeaxanthin/lutein is more hydrophilic than other carotenoids such as α- and β-carotene, lycopene, and β-cryptoxanthin; its mechanisms of action against carcinogenesis include induction of apoptosis, inhibition of angiogenesis, enhancement of gap junction intercellular communication, induction of cell differentiation, prevention of oxidative damage, and modulation of the immune system. Relevant studies are summarized in Table S6. The role of OFC was investigated by Batieha et al. in a case–control study, and they found that the risk of CC was significantly higher among women with lower levels of total serum carotenoids (OR 2.7; 95% CI, 1.1–6.4), α-carotene (OR 3.1; 95% CI, 1.3–7.6), and β-carotene (OR 3.1; 95% CI, 1.2–8.1) as compared to women in the upper tertiles [68]. The results from Harris et al. suggested that levels of β-carotene might have a weak inverse association with the risk of pre-invasive cancer but not with invasive cancer [69]. Instead, Palan et al. found significantly lower plasma levels of carotenoids (β-carotene, lycopene) in both women with pre-invasive and invasive CC [70]. Other positive evidence on the protective role of carotenoids against cervical dysplasia was provided by Nagata et al. (OR = 0.16, 95% CI 0.04–0.62 for the highest vs. lowest tertile of α-carotene; OR = 0.28, 95% CI 0.08–1.01 for the highest vs. lowest tertile of lycopene) [73]. Similar results were reported by Peterson et al., who showed an association between a low dietary intake of carotenoids and persistent infection (OR 2.4, 95% CI = 1.1–5.2), even if no association was found when plasma levels of micronutrients were considered [74]. Moreover, two prospective cohort studies observed a reduction in the risk of persistent HPV infection in women with higher plasma lycopene concentrations [75,76]. In agreement with the results mentioned above, a Brazilian case–control study showed that low serum levels of lycopene increased the risk of developing CIN3 [14,77]. Lastly, both Kanetsky et al. and Van Eenwyk et al. found that lycopene may be protective in the early stages of cervical carcinogenesis [78,79]. On the contrary, evidence against the protective role of carotenoids was reported by a case–control study that found an increased risk of cervical dysplasia in women with a high intake of β-carotene (OR 2.31, 95% CI 1.27–4.19) [80]. In a randomized control study of women with cervical dysplasia, the same authors administered 10 mg of β-carotene daily for three months to cases and a placebo to the control group. They found no effect of β-carotene on the regression of dysplasia [81]. Similar results were reported in another randomized control trial, in which the risk of persistence of CIN at nine months was comparable between cases supplemented with 30 mg of β-carotene daily and controls (OR = 1.53, 95% CI 0.38–6.18) [82]. Regarding OCX, a prospective cohort study found that maintenance of high levels of zeaxanthin/lutein in non-smokers patients is advantageous for preventing CC [83]. Similar results regarding the protective effect of zeaxanthin/lutein were found in a nested case–control study of 433 women participating in the Ludwig-McGill HPV Natural History Study; the authors observed that women with transient HPV infection had a higher daily mean intake of lutein/zeaxanthin compared to women who persistently tested positive for HPV(249 vs. 296 μg; *p* = 0.03) [84]. Moreover, higher circulating levels of lutein/zeaxanthin appeared to be associated with a significant decrease in the clearance time of HPV infection [85] and a lower risk of cervical intraepithelial neoplasia (OR = 0.40, 95% CI 0.17–0.95) [86]. On the contrary, Potischman et al. found no significant difference between mean serum lutein levels among 387 cases of invasive CC and 670 controls [87].

To summarize, many studies support the protective role of higher serum levels of OFC against CC, cervical dysplasia, and persistent HPV infection [75,76,77,78,82,83,84]. Moreover, low dietary intake and low serum levels of OFC may be associated with a higher risk of persistent HPV infection [79,80,81]. However, other studies found that oral supplementation of OFC did not affect the regression of cervical dysplasia [86,87], and one study found that the risk of dysplasia was higher in women with a high intake of beta carotenoids [85]. Regarding OCX, most studies support their protective role in preventing CC and persistent infection [88,89,90,91]. Only one study found no differences in levels of OCX between CC patients and controls [92].

### 3.7. Vitamin A

Vitamin A is a fat-soluble vitamin, including retinol and its derivates (retinyl esters, retin aldehyde, and retinoic acid). Vitamin A cannot be synthesized in human metabolism and is obtained through diet. The American Dietary Guidelines recommend daily consumption of 700 mcg to avoid deficiency. Vitamin A is involved in cell differentiation, growth, and maintaining and promoting immunity. Vitamin A derivatives are fundamental for cell signaling and regulation of gene expression. Relevant studies are summarized in Table S7. Decades ago, a case–control study found significantly higher mean serum retinol levels among cases (patients with cervical dysplasia) compared to healthy controls (606.6 vs. 640.6 ng/mL, *p* = 0.04). However, in the same study, dietary intake levels of retinol were not different between the two groups [88]. Lehtinen et al. found an association between low levels of serum Vitamin A and HPV infection in a small study. Recently, Huang et al. reported a non-linear correlation (U-shaped curve) between dietary intake and risk of infection, assuming a beneficial role of vitamin A in preventing HPV infection [89]. Findings from a case–control study by Yeo et al. demonstrated that patients in the lower quartile of serum retinol had an increased risk of CIN 1 compared to women with adequate levels (OR = 2.3, 95% CI = 1.3–4.1) [90]. Instead, a Brazilian cross-sectional study found serum levels that were more frequently low in HSIL patients without reaching a statistical significance after multivariate analysis (*p* = 0.409) [91]. The relationship between CC and vitamin A intake was investigated in a Korean case–control study enrolling 144 cases [92]. The authors found a statistically lower mean dietary intake in cases compared to controls, even when the amount from supplements was considered (*p* = 0.003) [92]. In the subgroup analysis of Ghosh et al., they reported a reduced risk of CC with a higher intake of vitamin A, comparing the highest to the lowest tertiles (OR = 0.47, 95% CI = 0.30–0.73) [93].

To summarize, studies support the protective role of higher serum levels of retinol on cervical dysplasia and HPV infection [93,94]. Additionally, lower serum levels were found in patients with HSIL [95] and may be associated with an increased risk of CIN1 [96]. Moreover, a higher dietary intake seems to be associated with the risk of CC [93,97].

### 3.8. Vitamin C

Vitamin C (ascorbic acid) is an essential micronutrient implicated in many biological functions. The chemical structure, bioavailability, and activity are the same for the natural and synthetic forms. It contributes to cholesterol, tyrosine, and folic acid metabolism and collaborates with epigenetic regulation of DNA, redox homeostasis, regulation of transcription factors, and collagen formation. Vitamin C has been demonstrated to improve the immune response by increasing leukocyte mobilization, neutrophil activity, and phagocytosis processes. Many cut-offs for optimal vitamin C levels have been proposed, but most authors consider ≤ 23.99 µmol/L in the serum a deficiency [84,94]. Relevant studies are summarized in Table S8. Decades ago, Brocket al. found that a high intake of ascorbic acid (more than 280 mg/day) may provide a significant protective effect against in situ cervical carcinoma [96]. Subsequently, an American case–control study [95] and the case–control study by Herrero et al. [97] confirmed a reduced risk of invasive CC in the highest quartile of the included population compared to the lowest quartile of vitamin intake (odds ratio = 0.69; *p* = 0.003). Other positive evidence for the protective role of CC was provided by Ghosh et al. [98] (highest vs. lowest vitamin C intake: OR 0.52; 95% CI: 0.33 to 0.80; *p* < 0.01) and Van Eenwyk et al. [79] (highest vs. lowest quartile for vitamin C intake: OR 0.20; 95% CI: 0.10 to 0.50; *p* < 0.005). The results from a Thailand case–control study enrolling 134 cases of invasive CC and 384 controls reported a trend favoring the protective effect of high vitamin C intake without reaching significance [99]. The association between ascorbic acid and HPV infection was investigated in a nested case–control study by Giuliano et al. [84], who found a decreased persistence of type-specific HPV infection in 12 months in the women with the highest intake (adjusted odds ratio 0.50; CI 95% 0.27–0.92). In an Italian cross-sectional study, 251 women with normal cervical cytology were evaluated concerning their HR-HPVs status. The authors found a negative association between dietary intake and infection [39]. First, Basu et al. [100] investigated the role of vitamin C serum levels in a small cohort of 45 women with cervical dysplasia. Naidu et al. [35] reported a significantly reduced plasma concentration of vitamin C in patients with CC compared to healthy controls. More recently, a cross-sectional study enrolling 2147 patients demonstrated a U-shaped relationship between serum vitamin C and HPV infection status [94]. Ascorbic acid was negatively correlated to infection but exclusively in the 25–59 age range, with the lowest risk reported for an approximate plasma level of 69 μmol/L [94].

To conclude, there is consistent evidence regarding the protective role of high dietary intake of ascorbic acid against CC and HPV persistent infections [35,40,84,89,100,101,102]. According to these results, lower serum levels of vitamin C have been found in patients with CC compared to controls [36], and a lower risk of HPV infection has been associated with higher serum levels of ascorbic acid [99].

### 3.9. Vitamin D

Vitamin D is commonly known for its role in calcium metabolism and bone remodeling. However, growing evidence demonstrates a role in cell growth modulation, neuromuscular and immune functions, anti-inflammatory activity, and glucose metabolism [103]. The synthesis of vitamin D begins in the bowel epithelium with pro-vitamin D3. Subsequently, in the skin, the ultraviolet radiation activates it into the pre-vitamin D3 form, which isomerizes to cholecalciferol [101]. Relevant studies are summarized in Table S9. Regarding the association between vitamin D intake and CCC, a case–control study among Japanese women found an inverse association between vitamin D intake and invasive cervical neoplasia risk (lowest quartile vs. highest quartile of vitamin D intake: OR 0.64, 95% CI 0.43–0.94). Regarding CIN3, in contrast, a protective effect of calcium and vitamin D intake was not demonstrated [102]. Other positive evidence regarding the protective effect of vitamin D intake comes from the randomized controlled trial performed by Vahedpoor et al. [104]. In this trial, 58 women diagnosed with CIN1 were randomly allocated into two groups to receive 50,000 IU vitamin D3 supplements or a placebo every two weeks. After six months, more women in the vitamin D group showed regression of CIN 1 compared to the placebo group (84.6% vs. 53.8%, *p* = 0.01) [104]. A subsequent RCT from the same authors found a beneficial effect on CIN 1–2–3 recurrence after LEEP with the same regimen of vitamin D3 supplementation (recurrence rate 18.5% vs. 48.1%vitamin D vs. placebo; *p* = 0.02). However, the difference in recurrence rate became non-significant after the exclusion of CIN1 cases [105]. On the other hand, treatment with vitamin D vaginal suppositories (12500 IU, three nights/week for six weeks) demonstrated anti-dysplastic effects against CIN1, but it showed no effect on CIN2 [106]. Regarding the association between serum levels of vitamin D and the risk of HPV infection, Özgü et al. reported a statistically significant difference in 25-OH Vitamin D3 levels (8.2891 vs. 11.4262 IU/mL, respectively; *p* = 0.009) between HPV-DNA positive women and the control group [107].

To summarize, higher vitamin D dietary intake and vitamin D supplementation (oral and vaginal) have been associated with a reduced risk of CC, higher CIN1 regression rate, and reduced risk of recurrence of dysplastic lesions after the LEEP procedure [105,107,108,109]. Moreover, significantly lower serum vitamin D levels have been found in patients with HPV infection compared to controls [110].

### 3.10. Vitamin E

Vitamin E is a lipid-soluble vitamin, presenting eight natural isoforms (α, β, γ, δ isoforms of tocopherol and α, β, γ, δ isoforms of tocotrienol). The anticancer activity of vitamin E is primarily due to its antioxidant, anti-inflammatory, antiproliferative, anti-angiogenic, and immune-modulatory functions [108]. α-Tocopherol is the main form of vitamin E in animals and carries the highest antioxidant activity [109]. Relevant studies are summarized in Table S10. Ghosh et al. found that women with a higher dietary intake of vitamin E carry a significantly lower risk of CC (OR 0.44; 95% CI 0.27–0.72 for the highest vs. lowest tertiles of vitamin E intake) [93]. Recent evidence showed that higher serum levels of vitamin E were associated with a reduced risk of CC [111]. In particular, a Chinese case–control study found a significant inverse association between α-tocopherol serum levels and the risk of CC (*p* = 0.002) [110]. Other positive evidence for the association between low plasma levels of tocopherols and the risk of cervical intraepithelial neoplasia/CC comes from the two cross-sectional studies by Palan et al. [112,113]. With regard to pre-invasive cervical lesions, a Brazilian case–control study observed that increasing serum concentrations of a- and γ-tocopherols were inversely associated with CIN3 risk (adjusted OR: 0.36, 95% CI, 0.18–0.74 for the highest vs. lowest quartile of a-tocopherol; adjusted OR: 0.51, 95% CI, 0.28–0.91 for the highest versus lowest tertile of γ-tocopherol) [14]. Similarly, Goodman et al. had previously found that the risk of squamous intraepithelial lesions was higher in patients with low plasma levels of α-tocopherol (OR 0.3, 95% CI 0.1–0.8 for the highest vs. lowest quartile) [114]. Lastly, the Ludwig–McGill cohort study reported that α- and γ-tocopherol may be protective against the persistence of non-oncogenic HPV infections [115].

To conclude, evidence suggests the protective role of a higher intake and high serum levels of vitamin E against CC and the risk of dysplasia [14,98,115,116,117,118]. Moreover, α- and γ-tocopherol may be protective against the persistence of non-oncogenic HPV infections [115].

### 3.11. Vitamin K

Vitamin K is a group of fat-soluble organic chemical compounds occurring in nature in two forms: vitamin K1, produced by green plants; vitamin K2, a series of compounds (menaquinones) produced generally by bacteria, except for menaquinone-4 which is produced in the human body. Vitamin K3 (menadione) is of synthetic origin [116]. The potential anticancer functions of vitamin K include inhibition of proliferation, induction of differentiation, inhibition of the potential for metastasis, and induction of autophagy or apoptosis [117]. Relevant results are summarized in Table S11. A recent Chinese cohort study administered a food frequency questionnaire to 218 randomly selected women. After adjusting for several confounders, the authors found that low dietary intake of vitamin K was associated with a higher risk of CIN 2–3 (second versus fourth quartile of vitamin K: OR = 1.60, 95% CI 1.05–2.44) [56]. Lastly, a cross-sectional investigation from the NHANES database among 13,447 participants found a non-linear relationship between vitamin K intake and the risk of HPV infection, with an inflection point at 14.03 mcg. The results of this study suggest that a dietary vitamin K intake of more than 14.03 mcg may reduce the risk of HPV infection. On the contrary, HPV subtypes were not associated with vitamin K intake [118].

To summarize, only a few studies are currently available regarding the association between vitamin K and the risk of CC. These studies support the protective role of vitamin K against the risk of HPV infection and CIN 2 and CIN 3 lesions [56,118].

## 4. Limitations of the Study

Nonetheless, this study has several limitations: most findings are derived from epidemiological or retrospective studies, and only a few randomized controlled trials are available in the literature resulting in low-quality results. In retrospective studies, possible biases may be due to selective recall or changes in metabolism and food habits after disease diagnosis. Moreover, regarding dietary intake, a non-negligible part of data derived from food frequency questionnaires was exposed to methodological biases and difficulties in accurately obtaining patient diet intake information. Moreover, the estimation of micronutrient amounts from food consumption does not consider food preparation, food processing, the freshness of the product, and bioavailability.

## 5. Conclusions

In the current study, we reported that different micronutrients might show a potential protective role against CC by intervening in different stages of the natural history of HPV infection, development of cervical dysplasia, and invasive disease.

Therefore, considering the design of included studies, we may speculate on the possible association. Still, the mechanisms through which micronutrients influence the infective and carcinogenic processes remain unclear, as well as concluding that the supplementation of specific micronutrients is effective against HPV-persistent infection or pre-neoplastic lesion progression is impossible. Moreover, studies in the literature did not allow us to compare the impact of dietary intake on serum levels, often reporting discordant results.

Based on the available evidence concerning the role of micronutrients, vitamins, and minerals on HPV infection and its progression to invasive CC, healthcare providers should be aware of the literature evidence and incorporate it in the counseling with other habit indications. However, further research, especially RCTs and prospective cohort studies, is mandatory to provide better evidence, and would clarify the biological mechanisms through which micronutrients may be protective against HPV infection and demonstrate if specific supplementations are effective.

## Data Availability

Data sharing not applicable.

## Supplementary Materials

The following supporting information can be downloaded at: https://www.mdpi.com/article/10.3390/healthcare11111652/s1, Table S1: summary of relevant studies on calcium; Table S2: summary of relevant studies on zinc; Table S3: summary of relevant studies on iron; Table S4: summary of relevant studies on selenium; Table S5: summary of relevant studies on folate and vitamin B12; Table S6: summary of relevant studies on carotenoids; Table S7: summary of relevant studies on vitamin A; Table S8: summary of relevant studies on vitamin C; Table S9: summary of relevant studies on vitamin D; Table S10: summary of relevant studies on vitamin E; Table S11: summary of relevant studies on vitamin K.
