# Supplementary material for: The Role of Micronutrients in Human Papillomavirus Infection, Cervical Dysplasia, and Neoplasm
Source: Healthcare (Basel). 2023 Jun 5;11(11):1652. doi: 10.3390/healthcare11111652 (PMC10252691; doi:10.3390/healthcare11111652)
Supplement: Supplementary file 1 [file healthcare-11-01652-s001.zip › healthcare-2384166-supplementary.pdf]

Table S1: summary of relevant studies on calcium

Author, year	Country	Design	Sample size, groups	Rational	Main findings
He, 2020	China	Cross sectional study	- 13475 participants from NHANES<br>- evaluation of dietary calcium	Association between dietary calcium and HPV infection status and HPV sub-type	- Calcium intake was not independently associated with HPV infection status or HPV sub-type.<br>- After converting dietary calcium intakes with the $\log_2$ function, a saturated effect was found: at the range of 3.32–9.78 of $\log_2$ calcium intake, a unit increase in calcium intake was associated with a 17% decrease of risk of HPV infection. After this range, no further decrease in risk of HPV infection could be observed.
Hwang, 2010	Korea	Cross sectional study	328 HPV-positive patients:<br>- Group 1: 166 HPV positive women without evidence of CIN<br>- Group 2: 90 CIN 1 + 72 CIN 2-3	Effects of dietary supplements on HR-HPV infection and cervical carcinogenesis	Dietary supplement including calcium significantly associated with a lower risk of CIN 2-3 (OR, 0.21; 95% CI, 0.08-0.50).
Liu, 1993	USA	Case-control study	- Group 1: 257 women with cervical dysplasia (cases)<br>- Group 2: 133 women negative at cytologic and colposcopic evaluation (controls)<br>24h dietary recall questionnaire to assess nutritional intake	Association between dietary intake and cervical dysplasia	No association between calcium intake and cervical dysplasia, with a multivariate-adjusted OR for the highest to lowest level of intake of 0.9 (95% CI 0.4–2.1)
Sengupta, 1989	India	Cross sectional study	- Group 1 (n 14): cervical carcinoma and pre-menopausal state<br>- Group 2 (n 14): cervical carcinoma and post-menopausal state<br>- Group 3 (n 14): healthy and pre-menopausal state<br>-Group 4 (n 14): healthy and post-menopausal state	Levels of serum calcium and cervical cancer state	Levels of serum calcium significantly higher in cervical cancer patients compared to controls:<br>- group 3 vs group 1: 10.44 mg/dL vs 14.41 mg/dL ($p<0.01$)<br>- group 4 vs group 2: 10.97 mg/dL vs 17.46 ($p <0.01$)

HPV: human papilloma virus; OR: odds ratio; CI: interval of confidence; NHANES: National Health and Nutrition Examination Survey; CIN: cervical intraepithelial neoplasia

Table S2: summary of relevant studies on zinc

Author, year	Country	Design	Sample size, groups	Rational	Main findings
Ayatollhai, 2022	Iran	RCT	- Group 1: 40, HPV test pos, LSIL<br>- Group 2: 40, controls (no treatment)<br>- Intervention: 220 mg zinc/12h for 3mo vs no treatment	Persistence of HPV infection and progression from baseline cytology	Group 1: persistence infection (OR = 0.130) (CI 95% 0.04-0.381; p <0.001) and progression 0.301 (95% CI 0.777-0.116; p =0.012
Xiao, 2022	China	Cross sectional study	- 4628 women from the NHANES<br>- Semi-quantitative food frequency questionnaire	Dietary intake of Zn and risk of HrHPV infection	Highest with vs lowest quartiles of Zn intake:OR f0.72 (95% CI, 0.54–0.98) for hrHPV infection.
Barchitta, 2020	Italy	Cross sectional study	- 251 women with normal citology<br>- Semi-quantitative food frequency questionnaire	Dietary intake of Zn and risk of HrHPV infection	Higher zinc intake have lower odds of infection with HR-HPVs (OR = 0.46; 95% CI = 0.27–0.80; p-value = 0.006)
Kim, 2011	Korea	Comparative study	- Group 1: 76, HPV test pos, LSIL<br>- Group 2: 118, controls (no treatment)<br>- Intervention: preparation of zinc citrate solution (0.5 mM) was administered topically 2/we for 3 mo vs no treatment	Impact of intravaginal zinc on persistency rate of HPV and cervical cytology regression	-Higher rates of HPV clearance in Gropup 1 (64.47%) vs Group 2 (25.51%) (p-value <0.001<br>-Reduced risk of persistent HPV in Group 1 (OR 0.079 (95% CI 0.039–0.165, -value<0.001).<br>- After 12 we, LISL lesions, 44.6% in Group 1 normalized cytologyvs21.4% of controls in Group 2 (p-value= 0.182)
Naidu,2007	India	Case control study	- 120 cases of CC vs 30 controls	Serum zinc level and risk of CC	Serum zincdecreased in cases compared to controls (p<0.001)
Cunzhi, 2002	Cina	Case control study	- 40 cases of CC vs 50 controls	Relationship between serum trace elements and the incidence of cervical cancer	-Serum zinc is lower in cases compared to controls (p<0.001)<br>- In cervical tissue zinc is lower than normal tissue (p<0.001)
Chen, 1990	Taiwan	Case control study	99 cases of CC vs 50 controls	Serum zinc level variation in CC	Zinc level decreased in stage III or IV and recurrent patients (67.2 +/- 16.6 and 70.4 +/- 17.2 micrograms/dl)
Grail, 1986	Scotland	Case control study	110 CIN vs 9 cc vs 21 controls	Serum zinc level in CIN, CC and controls	Mean serum zinc levels decreased in LSIL (0.81 mg/l) and in invasive carcinoma (0.73 mg/l) compare to controls (p<0.005)

RCT: randomized controlled trial; HPV: human papilloma virus; HrHPV: high risk human papilloma virus; LSIL: low squamous intraepithelial lesion; OR: odds ratio; CI: interval of confidence; NHANES: National Health and Nutrition Examination Survey; we: week; mo:months; CC: cervical cancer.

Table S3: summary of relevant studies on iron

Author, year	Country	Design	Sample size, groups	Rational	Main findings
Siegel, 2012	USA	Cohort study	327 women enrolled in the Ludwig-McGill Cohort study	Ferritin serum level and the Incident HPV clearance rates (any-type, oncogenic HPV, non-oncogenic HPV, and HPV-16)	- Median duration of HPV infections did not significantly differ by iron status (adjusted HR 0.88, 95%CI 0.72, 1.07).<br><br>- Ferritin levels above the median were less likely to clear an incident oncogenic HPV (adjusted HR=0.73; 95%CI 0.55–0.96) and HPV-16 infections (adjusted HR=0.29; 95%CI 0.11–0.73).<br><br>- Women with enriched iron stores (ferritin ≥120ug/L) were less likely to clear incident oncogenic HPV infections compared to those with low-levels of iron (<20ug/L) (adjusted HR=0.34; 95%CI 0.15–0.81)<br><br>- No significant association between ferritin at adequate or enriched levels and clearance of incident non-oncogenic HPV infections
Cunzhi, 2003	China	Case control study	- 40 cases of patients with cervical cancer<br>- 30 cases of uterine myoma<br>- 50 healthy subjects	Tissue and serum levels of iron	- Iron concentration in cervical cancer tissue was significantly higher than in non-lesion tissue (p<0.005)<br>- Iron serum concentration significantly lower in patients with cervical cancer compared to healthy subjects (p<0.001)
Ito, 1980	Japan	Case control study	98 patients withuntreated cervical cancer<br>52 healthy controls	Serum ferritin levels and prognosis of cervical cancer	- In 98 patients with untreated cervical cancer, 50 (51%) had elevated levels of serum ferritin.<br><br>- Among 36 patients who were surgically treated, 12 of 16 patients (75%) with ferritin levels above normal and 3 of 20 patients (15%) with levels below 108 ng/ml had parametrial invasion and/or lymph node metastasis.<br><br>- Among 21 patients with elevated levels of ferritin before treatment, levels decreased to the normal range 4 weeks after treatment in many patients, but the development of elevated levels thereafter was closely associated with a poor prognosis.

HPV: human papilloma virus; OR: odds ratio; aOR: adjusted odds ratio; HR: hazard ratio; CI: interval of confidence; CIN: cervical intraepithelial neoplasia; SILs: squamous intraepithelial lesions

Table S4: summary of relevant studies on selenium

Author, year	Country	Design	Sample size, groups	Rational	Main findings
Xiao, 2022	China	Cross sectional study	4628 women from the NAHNES<br>Se intakes were assessed from two 24-h diet recalls	Relationship between selenium dietary amounts and hrHPV infection	No significant association between intakes of selenium and hrHPV infection in multivariate analysis
Obhielo, 2019	Nigeria	Case control study	- 45 women with CIN (cases)<br>- 45 women (age matched controls) with normal cervical cytology	Relationship between serum level of selenium and CIN	- Mean serum selenium levelin cases was 52.32 ± 20.46µg/L vs 59.49 ± 17.20µg/L in the control group. This difference was not statistically significant.<br>- A statistically significant difference in the mean value of serum selenium observed between the controls and histological subgroups within the cases (CIN I, CIN II, and CIN III) using the ANOVA test (p= 0.021).<br>- A statistically significant difference between controls (with normal cervix) and CIN 3 (p value = 0.016).
Karamali, 2015	Iran	RCT	58 women with CIN1 randomly assigned to:<br>-Group 1 (n 28): 200 mg of Se (form: Se yeast) per day for 6 months<br>- Group 2 (n 28): placebo for 6 months	effects of long-term Se administration on the regression of CIN1	A greater percentage of women in the Se group had regressed CIN1 (88·0 v. 56·0 %; P = 0·01) compared with those in the placebo group
Cunzhi, 2003	China	Case control study	- 40 cases of patients with cervical cancer<br>- 30 cases of uterine myoma<br>- 50 healthy subjects	Tissue and serum levels of selenium	- Selenium concentration in cervical cancer tissue was significantly higher than in non-lesion tissue (p<0.001)<br>- Selenium serum concentration significantly lower in patients with cervical cancer compared to healthy subjects (p<0.001)
Sundström, 1986	Finland	Cross sectional study	- 25 cervical cancer patients<br>- 32 control women	Serum selenium concentration	Selenium plasma concentration in cervical cancer patients was significantly lower than in control subjects (0.97 ± 0.06 µmol/L vs 1.26 ± 0.03µmol/L, p<0.001)

RCT: randomized controlled trial; HPV: human papilloma virus;hrHPV: high risk human papilloma virus infection;NHANES: National Health and Nutrition Examination Survey; OR: odds ratio; CI: interval of confidence; CIN: cervical intraepithelial neoplasia.

Table S5: summary of relevant studies on folate and vitamin B12

Author, year	Country	Design	Sample size, groups	Rational	Main findings
Li, 2018	China	Case control study	80 normal cervix vs 55 CIN1 and55 CIN2/3 and 64 CC	influences of folate on FHIT gene methylation and expression in the progression of cervical cancerization	- The proliferation inhibition rate, apoptosis rate, and FHIT protein and mRNA expression levels increased with rising concentrations of folate<br>-Serum folate significantly decreased in CIN and CC groups (H=43.68, P<0.001)
Sabihi, 2022	Iran	RCT	Group 1: 30 CIN2/3; 5mg/day folate x 12 we<br>Group 2: 30 controls, placebo	impact of folate supplementation on recurrence of CIN2/3	-non-significant decrease in recurrence of CIN2/3 in Group 1 in comparison with Group 2 (3.3% vs. 16.7%, p= 0.08)
Zhao, 2016	China	Cross sectional study	20000 women; 247 LSIL, 125 HSIL and 877 controls	effect of folate status on CIN progression and relationship with hrHPV	- Increased CIN correlated with higher rates of hrHPV infection and lower levels of serum folate<br>-HSIL had a significantly lower folate status than those with LSIL and normal cervical histology (p=0.02)
Piyathilake, 2014	India	Cross sectional study	315 HPV 16 positive women diagnosed with either CIN 2 or ≤ CIN 1.	association of plasma concentrations of folate and vitamin B12 on the degree of HPV 16m	- higher plasma folate and higher HPV 16m or higher plasma vitamin B12 and higher HPV 16m were 75% (P<0.01) and 60% (P=0.02) less likely to be diagnosed with CIN 2<br>- With a tertile increase in the plasma folate or vitamin B12, there was a 50% (p=0.03) and 40% (p=0.07) increase in the odds of a higher degree of HPV 16m
Piyathilake, 2010	India	Cross sectional study	724 women screened for HPV	associations between serum concentrations of folate and vitamin B12 and hrHPV infections	- higher concentrations of serum folate (6 ng/mL) and vitamin B12 (356 pg/mL) were at lower risk of being positive for hrHPVs compared to those with serum folate lower than 6 ng/mL and serum vitamin B12 356pg/mL (odds ratio = 0.26; 95% confidence interval: 0.08–0.89; p= 0.03)
Bai, 2014	China	Cross sectional study	109 normal cervix, 101 CIN, 101 CC	interactions by examining serum folate and DNA methylation of tumor suppressor gene FHIT and HPV 16 in women with normal cervix, CIN or CC	-ORs only with FHIT methylation (OR=11.47) or only with HPV 16 positive (OR=4.63) or with serum folate level lower than 3.19ng/ml (OR=1.68) in CC group were all higher than the control status of HPV 16 negative and FHIT unmethylation and serum folate level more than 3.19ng/ml (OR=1)
Raghasuda, 2012	India	Case control study	136 control subjects vs 92 LSIL and 94 invasive CC	Association of serum folate and vit B12 with LSIL and C	-The risk estimates observed for B12 became prominent only when there was a deficiency in serum folate levels [LSIL OR:14.9 (95% CI: 2.65 to 84.4); CC–OR = 8.72 (95% CI = 1.55 to 48.8)] or when MTHFR A1298C polymorphic variant was present [LSIL OR = 9.8 (95% CI = 2.61 to 36.7); CC–OR = 10.0 (95%CI = 2.5 to 39.3)]
Abike, 2010	Turkey	case control study	- 122 women screened for HPV	Association of serum folate and vit B12 with HPV infection persistence and CIN	-all cervical dysplasia groups, folate levels were lower in HPV-positive patients than in HPV-negative patients (P<0.05)

Asemi, 2015	Iran	RCT	Group 1: 29 CIN1 5 mg/d folate x 6 mo<br>Group 2: 29 CIN1 placebo	Effects of long-term folate supplementation on regression and metabolic status of patients with CIN1	A greater percentage of women in Group 1 regressed compared to Group 2(83.3 versus 52.0%,p= 0.019)
Wang, 2006	China	case control study	111 CC vs 111 controls	effects of dietary intake of folate on cervical carcinogenesis and HPV16 infection	serum folate in CC cases (1.79 ng/ml +/- 1.42 ng/ml) was significantly lower than that in controls(2.59 ng/ml +/- 2.81 ng/ml) (p<0.05)
Hernandez, 2003	USA	case control study	-214 LSIL/HSIL vs 217 controls	Association of dietary intake and CIN	inverse association between total folate intake and premalignant cervical lesions, especially for HSIL (p=0.04)

RCT: randomized controlled trial; Yo: years old; CC: cervical cancer; CIN: cervical intraepithelial neoplasia; CI: confidence interval; OR: odds ratio; RR: relative risk; hrHPV: high risk human papilloma virus; We: week; HPV 16m: HPV 16 methylation; MTHFR: methylenetetrahydrofolate reductase; LSIL: low grade squamous intraepithelial; HSIL: high grade squamous intraepithelial

Table S6: summary of relevant studies on carotenoids

Author, year	Country	Design	Sample size, groups	Rational	Main findings
Kanetsky, 2015	USA	Case control study	-32 women with cervical dysplasia (12 diagnosed with CIN 1, 10 with CIN 2, and 9 with CIN 3)<br>- 113 controls<br>Food frequency questionnaire and blood samples.	Dietary intake of carotenoids and risk of cervical dysplasia	Women in the upper tertile of lycopene intake were one-third (OR = 0.32, 95% CI = 0.8-1.3) and one-fourth (OR = 0.24, 95% CI = 0.05-1.2) as likely, respectively, to have dysplasia as women in the lower tertile. Elevated levels of serum lycopene also suggested some protection against dysplasia. Results were not significant at α = 0.05
Fujii, 2012	Japan	Prospective study	- 391 patients with CIN 1-2<br>- serum micronutrient concentrations and a questionnaire about dietary intake	serum concentrations and dietary intake of micronutrients and regression/progression of low-grade cervical abnormalities.	In non-smoking subjects, regression of CIN significantly associated with serum levels of zeaxanthin/lutein (HR 1.25, 95% CI 0.78–2.01, p = 0.024). In progression subjects, a significant protective effect against progression to CIN3 in individuals with a medium level of serum β-carotene (HR 0.28, 95 % CI 0.11–0.71), although any protective effect from a higher level of serum β-carotene was weaker or abolished (HR 0.52, 95 % CI 0.24–1.13)
Peterson, 2010	USA	Prospective study	-120 women: persistent oncogenic HPV infection<br>- 141 women: persistent non oncogenic HPV infection	Association between dietary and serum levels of micronutrients and persistence of HPV infection	- No significant association between serum carotenoid levels and persistent infection with any type HPV.<br>-A greater than twofold increased risk of persistent infection with any type HPV was observed among women with low carotenoid status using dietary intake nutrients (p= 0.03).
Tomita, 2011	Brazil	Case control study	-231 confirmed cases of CIN3<br>- 453 controls<br>Food frequency questionnaires and serum levels	Association between smoke, dietary and serum levels of micronutrients and CIN 3	Low serum levels of lycopene resulted in an increased risk of developing CIN3 (OR 1.88, 95% CI 1.14-3.08).
Goodman, 2007	USA	Cohort study	-189 incident oncogenic HPV infections among 122 women	Associationbetween carotenoid concentrations with the clearance of incident cervical HPV infection	Higher circulating levels of lutein/zeaxanthin, cryptoxanthin, lycopene, carotenes and total carotenoids associated with a significant decrease in the clearance time of HPV infection, particularly during the early stages of infection (< 120 days)
Giuliano, 2003	USA	Case control study	-433 women: 248 transient HPV infections and 185 had persistent HPV infections<br>food frequency questionnaire	association betweendietary intake and persistence of HPV infection	Risk of persistent HPV infection was lower among women reporting intake values of β-cryptoxanthin and lutein/zeaxanthin in the upper 2 quartiles<br>compared with those reporting intake in the lowest quartile
Sedjo, 2003	USA	Prospective study	84 women with at least 1 oncogenic HPV infection at baseline	Plasma levels of carotenoids and clearance of HPV infection	The likelihood of clearing an oncogenic HPV infection was significantly higher with increasing levels of trans-lycopene (p for trend, 0.025) and cis-lycopene (p for trend, 0.010).<br>The aOR of the highest tertiles of trans- and cis-lycopene was 2.79 (95% CI 1⁄4 1.17–6.66) and 2.92 (95% CI 1⁄4 1.28–6.63) compared with the lowest tertiles.
Sedjo, 2002		Prospective study	1042 women	role of carotenoids on HPV	A 56% reduction in HPV persistence risk was observed in

			food frequency questionnaire + plasma level of micronutrients	persistence and regression	women with the highest plasma cis-lycopene concentrations compared with women with the lowest plasma levels (aOR 0.44; 95% CI, 0.19–1.01).
Schiff, 2001	USA	Case control study	-81 cases: biopsyproven CIN 2 e CIN 3<br>- 160 controls	Association between serum carotenoids and CIN	Increasing levels of alpha-carotene, β-cryptoxanthin, and lutein/zeaxanthin were associated with decreasing risk of CIN 2-3. The highest tertiles of β-cryptoxanthin (OR 0.39, 95% CI 0.17–0.91) and lutein/zeaxanthin (OR 0.40, 95% CI 0.17–0.95) were associated with the lowest risk of CIN
Nagata, 1999	Japan	Case control study	-152 women with cervical dysplasia<br>- 152 controls with normal cervical cytology	Plasma levels of micronutrients and risk of cervical dysplasia	Significantly lower serum levels of α-carotene, β-carotene and lycopene were observed in cases.
Palan, 1996	USA	Case control study	- 140 women with CIN or cervical cancer<br>-95 controls	Plasma carotenoids levels and cervical cancer/dysplasia	The mean plasma levels of carotenoids were significantly lower in women with CIN and cervical cancer
Romney, 1996	USA	RCT	69 omen:<br>- cases: daily oral supplementation of 30 mg of βcarotene for 9 months<br>- controls: placebo for 9 months	evaluate the efficacy of β-carotene to cause regression of CIN.	The β-carotene and placebo groups did not differ in risk for having CIN at 9 months (OR = 1.53, CI 0.38–6.18)
Batieha, 1993	USA	Case control study	- 50 cases of cervical cancer<br>-100 controls	Relationship between serum micronutrients and risk of cervical cancer.	The mean serum levels of total carotenoids, alpha-carotene, β-carotene, cryptoxanthin, and lycopene were lower among cases than controls. When examined by tertiles, the risk of cervical cancer was significantly higher among women in the lower tertiles of total carotenoids (OR 2.7; 95% CI, 1.1-6.4), alpha-carotene (OR 3.1; 95% CI, 1.3-7.6), and β-carotene (OR 3.1; 95% CI, 1.2-8.1) as compared to women in the upper tertiles.
De Vet, 1991	Netherlands	Case control study	- 257 caseswith cervical dysplasia who took 10 mg supplement of β-carotene/die<br>- 705 controls<br>- food frequency questionnaire	effects of the dietary intake of β-carotene on cervical dysplasia.	Increased risk of cervical dysplasia for women with a high intake of β-carotene (> 3.5 mg/die vs < 1.5 mg/die): OR 2.31; 95% CI:1.27-4.19.
De Vet, 1991	Netherlands	RCT	137 cases with cervical dysplasia<br>Treated with 10mg of β-carotene daily for 3 months<br>- 141 controls with cervical dysplasia treated with placebo for 3 months	Effect of β-carotene of regression/progression of cervical dysplasia	No effect of β-carotene on the regression percentages was observed. A secondary analysis, in which the effect of the total intake of β-carotene (diet + medication) on the regression percentages of cervical dysplasia was studied, did not show a positive effect either
Potischman, 1991	USA	Case control study	- 387 cases of invasive cervical cancer<br>- 670 controls	Association between serum levels of micronutrients and risk of invasive cervical cancer	No association between serum level of lutein and invasive cervical cancer
VanEenwyk, 1991	USA	Case control study	-102 cases with biopsy confirmed CIN 1, 2 or 3<br>- 102 controls	Association between CIN and serum and dietary carotenoids	OR for those in quartiles 3,2,1 compared to quartile 4 (highest) of serum lycopene were 3.5 (95% CI 1.1-11.5), 4.7 (95% CI 1.2-17.7) and 3.8 (95% CI 1.1-12.4), respectively.<br>aORfor higher vs lowest quartile of dietary lycopene were: 4.6 (1.1-19.7), 5.8 (1.6-21.3) and 5.4 (1.3-23.3).

Harris, 1986	UK	Case control study	-113 women with cervical cancer (32 invasive and 81 pre-invasive)<br>- 226 controls	Association between levels of β-carotene and cervical cancer	mean β-carotene levels were significantly reduced in women with pre-invasive disease compared with controls (221.3 vs 291.6ug/L, $p<0.05$)

RCT: randomized controlled trial; HPV: human papilloma virus; HR: hazard ratio; OR: odds ratio;aOR adjusted odds ratio; CI: interval of confidence; CIN: cervical intraepithelial neoplasia.

Table S7: summary of relevant studies on vitamin A

Author, year	Country	Design	Sample size, groups	Rational	Main findings
Huang, 2020	China	Cross sectional study	13412 women with available HPV test	Relation between vit.A intake and HPV infection	- curvilinear relationship between vit A and HPV infection (U-shaped curve)<br>- appropriate amount (95% CI: 0.9–1.0, <10.5 of log2 transformer, i.e., 1448.155 mcg) of dietary vit A beneficial to prevent HPV infection.
Eleuterio, 2014	Brasil	Cross sectional study	62 women with confirmed SIL diagnosis	serum levels of vit A in women diagnosis SIL	The level of Vit A more frequently low in HSIL, but with no statistical significance (p = 0.409)(OR: 2.26 ;0.33–15.59)
Ghosh, 2008	USA	Case control study	239 CC vs 979 controls	evaluation of different intakes of selected dietary nutrients and food groups and risk of cervical cancer	women in the highest vs. lowest tertiles of dietary reduced risk of cervical cancer with higher intakes of vit. A (OR = 0.47, 95% CI = 0.30–0.73)
Kim, 2010	Korea	Case control study	144 CC vs 288 controls	Relation between vit.A intake and CC	highest quartiles of dietary vitamin C intake had statistically significantly lower cervical cancer risks than those in the lowest quartiles for vitamin COR = 0.36 (CI = 0.18–0.69)
Yeo, 2000	USA	Case control study	326 controls vs 190 CIN1 and 112 CIN2/3	Relation between serum level of vit.A intake and CIN	subjects in the lowest serum vit.A quartile were at increased risk of CIN I compared with women in the highest quartile (OR = 2.3, 95% CI = 1.3–4.1) (p=0.001)
Shimizu, 1996	Japan	Case control study	137 cases with cervical dysplasia vs controls matched for age and screening date	Relation between serum and dietary Vit.A and the risk of cervical dysplasia	-Mean serum retinol levels were significantly lower among cases compared with controls (606.6 vs 640.6ng/mL. p 0.04)<br>- When examined by tertile, the risk of cervical dysplasia was significantly higher among women in the highest tertile of dietary vitamin A level (OR 2.45 95% CI 1.11-5.38).

Yo: years old; CC: cervical cancer; CIN: cervical intraepithelial neoplasia; CI: confidence interval; OR: odds ratio; RR: relative risk; HPV: human papilloma virus.

Table S8: summary of relevant studies on vitamin C

Author, year	Country	Design	Sample size, groups	Rational	Main findings
Zheng, 2022	China	Cross sectional study	2174 women, 18–59 years of age	associations between serum vitamin C levels and HPV infection	- serum vitamin C levels negatively associated with HPV infection in women≥25 yo, not under 25<br>- negative correlation between adequate vitamin C and HPV infection (OR 0.7, 95% CI: 0.52–0.94)
Barchitta, 2020	Italy	Cross sectional study	251 women with normal cervical citology	associations between serum vitamin C levels and Hr-HPV infection	Hr-HPV women reported lower intake of zinc compared to non-infected women (p<0.002
Ghosh, 2008	USA	Case control study	239 cases of CC vs 979 controls	impact of vit. C dietary intake and risk of CC	reduced risk for women in the highest vs. lowest tertilesof vit. C dietary intake (OR = 0.52, 95% CI =0.33–0.80)
Naidu, 2007	India	Case control study	120 CC vs 30 controls	associations between serum vitamin C levels and CC	decreased levelsof plasma Vitamin-C (p<0.001) in all the stages of CC patients compared with controls
Giuliano, 2003	USA	Nested case study	248 transient HPV infection; 185 persistent HPV infection	association betweendietary intake and persistence of HPV infection	decreased persistence of type-specific HPV infection in a period of 12 months in the women with highest intake (adjusted odds ratio 0.50; CI 95% 0.27–0.92)
Shannon, 2002	Thailand	Case control study	-134 invasive CC vs 384 controls<br>-50 CC in situ vs 125 controls	evaluate the possible effects of specificdietary factors on cervical carcinogenesis.	higher intake of vitamin C reducea risk of either in-situ or invasive cervical cancer
VanEewyk, 1992	USA	Case control study	100 CIN vs 102 controls	Association of vit. C dietary intake and CIN	reduced risk for women in the highest vs lowest quartile for vitamin C intake: OR 0.20; 95% CI: 0.10 to 0.50; p< 0.005
Herrero, 1992	Costa Rica	Case control study	748 invasive CC vs 1411 controls	Association between vit. C intake and risk of CC	decreasing risk observed for adequate vitamin C (OR = 0.69 for the highest vs.the lowest quartile; p= 0.003)
Ziegler, 1991	USA	Case control study	229 case in situ CC vs 502 controls	Association between vit. C intake and risk of CC	The highest risk among consumers in the Vit. C lowest quartile of intake(RR of 1.5)
Brock, 1988	Australia	Case control study	117 CC in situ vs 196 controls	Vit C dietary intake and risk of CC	High intake of vit C (>280 mg/day) seems to provide a substantial protective effect

Yo: years old; CC: cervical cancer; CIN: cervical intraepithelial neoplasia; CI: confidence interval; OR: odds ratio; RR: relative risk; HPV: human papilloma virus.

Table S9: summary of relevant studies on vitamin D

Author, year	Country	Design	Sample size, groups	Rational	Main findings
Vahedpoor, 2018	Iran	RCT	58 patients with CIN2-3 treated with LEEP randomly assigned to 2 groups:<br>- Group 1: 50,000 IU vitamin D3 (n:29) every 2 weeks for 6 months<br>- Group 2: placebo (n: 29) every 2 weeks for 6 months	Effects of vitamin D supplementation on the recurrence of CIN	The recurrence rate of CIN 1-2-3 was 18.5% and 48.1% in the vitamin D and placebo groups respectively (p = 0.02).<br>After excluding CIN1 cases of recurrence, the recurrence rate of CIN2-3 became nonsignificant (3.7% in the vitamin D group vs. 14.8% in the placebo group, p = 0.15).
Vahedpoor, 2017	Iran	RCT	-58 women diagnosed with CIN 1randomly assigned to 2 groups:<br>- Group 1: 50,000 IU vitamin D3 (n:29) every 2 weeks for 6 months<br>- Group 2: placebo (n: 29) every 2 weeks for 6 months	Effects of long-term vitamin D administration on regression of CIN1	Greater percentage of women in the vitamin D group had regressed CIN1 (84.6 vs. 53.8%, p = 0.01) than those in the placebo group.
Özgü, 2015	Turkey	Case control study	- 23 cases of HPV DNA positive women<br>- 62 controls	Serum level of 25-OH vitamin D3 and risk of HPV DNA positivity	-Mean of 25-OH Vitamin D3 levels of study and control groups were 8,0857 IU/ml and 11,4720 IU/ml respectively(p=0,009).
Schulte-Uebbing, 2014	Germany	Cross sectional study	-Group 1: 100 patients with chronical bacterial and/or fungal infection and no cervical dysplasia.<br>- Group 2: 100 patients with chronical bacterial and/or fungal infections and cervical dysplasia (50 with a CIN 1 and 50 with a CIN 2).<br>Intervention: vitamin D vaginal suppositories (12.500 IU, 3 nights a week, for 6 weeks).	Effects of vaginal vitamin D supplementation on cervical infections and cervical dysplasia	Group 1: 79% of the women had “less vaginal problems,” “less discharge” and “less problems with the sexual intercourse.”<br><br>Group 2: In the CIN 1 group, after six weeks of treatment, a goodantidysplastic effects was noted. No effect was evident on CIN 2.
Hosono, 2010	Japan	Case control study	- 405 incident cervical neoplasia (333 invasive cervical carcinomas + 72 CIN 3)<br>- 2025 controls<br>Food frequency questionnaires	Dietary intake of vitamin D and cervical neoplastic risk	- No association between vitamin D intake among CIN3 was evident (P for trend = 0.109).<br>- An inverse association between Vitamin D intake and cervical neoplastic risk (lowest vs highest quartile of vitamin D intake OR 0.64, 95% CI 0.43–0.94)

RCT: randomized controlled trial; HPV: human papilloma virus; LEEP: Loop Electrosurgical Excision procedure; OR: odds ratio; CI: interval of confidence; CIN: cervical intraepithelial neoplasia.

Table S10: summary of relevant studies on vitamin E

Author, year	Country	Design	Sample size, groups	Rational	Main findings
Guo, 2015	China	Case control study	- 458 cases of invasive cervical cancer<br>- 742 controls<br>- serum vitamin E levels	Serum levels and dietary intake of vitamin E levels and risk of cervical cancer	The OR for the highest (vs. lowest) quartile of serum vitamin E was 0.53 (95% CI = 0.37–0.74; *P* < 0.001).
Zhang, 2015	China	Case control study	- 158 incident cases of cervical cancer<br>- 200 controls	Plasma levels of a- and γ-tocopherols and risk of cervical cancer	Inverse associations between α-tocopherol concentrations and the risk of cervical cancer after adjusting for potential confounders (p 0.002).
Tomita, 2010	Brazil	Case control study	- 453 controls<br>- 4 groups of cases (CIN1: 140; CIN2: 126; CIN3: 231; invasive cancer: 108)	Serum and dietary intake of tocopherol and risk of CIN	Increasing serum concentrations of α- and γ-tocopherols were inversely associated with CIN3 risk; the aOR for the highest compared to the lowest quartile of α-tocopherol was 0.36 (95%CI, 0.18–0.74) and for the highest versus lowest tertile of γ-tocopherol was 0.51 (95%CI, 0.28–0.91) after adjusting for confounding variables.
Ghosh, 2008	USA	Case control study	- 239 cases diagnosed with squamous cell carcinoma of the cervix<br>- 979 patients with non neoplastic diagnosis<br>- self administered questionnaire	Association between dietary vitamin E intake and HPV infection status	Significant reduction of risk observed in women in the highest vs lowest tertiles of vitamin E intake (OR = 0.44, 95% CI = 0.27–0.72)
Siegel, 2006	USA	Cohort study	- 405 HPV positive women: 229 oncogenic and 176 non-oncogenic infections	Serum levels of tocopherol and HPV persistence	Midlevels of α-tocopherol were inversely associated with nononcogenic HPV persistent infection (aOR 0.28, 95% CI 0.14–0.57), while high levels were marginally associated (aOR 0.59, 95% CI 0.28–1.19).<br>Circulating levels of α- and δ-tocopherol in the middle or upper tertiles inversely associated with non-oncogenic HPV persistence (aOR 0.44, 95% CI 0.19– 0.97 and aOR 0.46, 95% CI 0.19–1.11, respectively).
Palan, 2004	USA	Cross sectional study	- study groups: 37 women with cervical intraepithelial neoplasia + 14 women with cervical cancer<br>- 21 controls	Plasma levels of α-tocopherol and α-tocopheryl quinone	Plasma levels of a-tocopherol and a-tocopheryl quinone were decreased significantly (P=0.012 and=0.005, respectively) in study groups compared with the control group
Palan, 2003	USA	Cross sectional study	- 48 controls without any history of abnormal Pap smears<br>- patients with histopathologically confirmed diagnoses of CIN 1: 98 patients; CIN 2: 49 patients, CIN 3: 10 patients and cervical cancer: 25 patients.	mean plasma levels of α-tocopherol and γ-tocopherol and risk of CIN	Mean plasma levels a- tocopherol and γ-tocopherol were significantly lower (P < 0.001, and < 0.001, respectively) in patients with various grades of CIN and cervical cancer compared with controls.
Goodman, 1998	USA	Case control study	- 147 cases of SILs and 191 controls<br>- plasma levels of α-tocopherol	Plasma levels of α-tocopherol and risk of SILs	The OR among women in the highest compared with the lowest quartile was 0.3 *(95%* CI, 0.1-0.8) for α-tocopherol.

HPV: human papilloma virus; OR: odds ratio; aOR: adjusted odds ratio; HR: hazard ratio; CI: interval of confidence; NHANES: National Health and Nutrition Examination Survey; CIN: cervical intraepithelial neoplasia; SILs: squamous intraepithelial lesions;

Table S11: summary of relevant studies on vitamin K

Author, year	Country	Design	Sample size, groups	Rational	Main findings
Jiang, 2022	China	Cross sectional study	- 13447 participants from NHANES<br>- evaluation of dietary vitamin K intake	Association between dietary vitamin K and HPV infection status	More than 14.03mcg of dietary vitamin K intake maybe reduce the risk of HPV-infection. HPV-subtype was not associated with vitamin K intake.
Wang, 2020	China	Cohort study	- 218 randomly selected subjects<br>- semi-quantitative food frequency questionnaires	Association between dietary nutrient intake and CIN risk	Low dietary vitamin K was associated with CIN2+ risk (second versus fourth quartile: OR = 1.60, 95% CI 1.05–2.44)

HPV: human papilloma virus; OR: odds ratio; aOR: adjusted odds ratio; HR: hazard ratio; CI: interval of confidence; NHANES: National Health and Nutrition Examination Survey; CIN: cervical intraepithelial neoplasia; SILs: squamous intraepithelial lesions
